# A Post-Hospital Home Exercise Program Improved Mobility but Increased Falls in Older People: A Randomised Controlled Trial

**DOI:** 10.1371/journal.pone.0104412

**Published:** 2014-09-02

**Authors:** Catherine Sherrington, Stephen R. Lord, Constance M. Vogler, Jacqueline C. T. Close, Kirsten Howard, Catherine M. Dean, Gillian Z. Heller, Lindy Clemson, Sandra D. O'Rourke, Elisabeth Ramsay, Elizabeth Barraclough, Robert D. Herbert, Robert G. Cumming

**Affiliations:** 1 The George Institute for Global Health, The University of Sydney, Sydney, New South Wales, Australia; 2 Neuroscience Research Australia, University of New South Wales, Randwick, New South Wales, Australia; 3 Northern Clinical School, The University of Sydney, Sydney, New South Wales, Australia; 4 Department of Aged Care and Rehabilitation, Royal North Shore Hospital, St Leonards, New South Wales, Australia; 5 Prince of Wales Clinical School, University of New South Wales, Randwick, New South Wales, Australia; 6 School of Public Health, The University of Sydney, Sydney, New South Wales, Australia; 7 Department of Health Professions, Faculty of Human Sciences, Macquarie University, Macquarie Park, NSW, Australia; 8 Department of Statistics, Macquarie University, Macquarie Park, New South Wales, Australia; 9 Discipline of Occupational Therapy, Faculty of Health Sciences, The University of Sydney, Lidcombe, New South Wales, Australia; University of Glasgow, United Kingdom

## Abstract

**Background:**

Home exercise can prevent falls in the general older community but its impact in people recently discharged from hospital is not known. The study aimed to investigate the effects of a home-based exercise program on falls and mobility among people recently discharged from hospital.

**Methods and Findings:**

This randomised controlled trial (ACTRN12607000563460) was conducted among 340 older people. Intervention group participants (n = 171) were asked to exercise at home for 15–20 minutes up to 6 times weekly for 12 months. The control group (n = 169) received usual care. Primary outcomes were rate of falls (assessed over 12 months using monthly calendars), performance-based mobility (Lower Extremity Summary Performance Score, range 0–3, at baseline and 12 months, assessor unaware of group allocation) and self-reported ease of mobility task performance (range 0–40, assessed with 12 monthly questionaries). Participants had an average age of 81.2 years (SD 8.0) and 70% had fallen in the past year. Complete primary outcome data were obtained for at least 92% of randomised participants. Participants in the intervention group reported more falls than the control group (177 falls versus 123 falls) during the 12-month study period and this difference was statistically significant (incidence rate ratio 1.43, 95% CI 1.07 to 1.93, p = 0.017). At 12-months, performance-based mobility had improved significantly more in the intervention group than in the control group (between-group difference adjusted for baseline performance 0.13, 95% CI 0.04 to 0.21, p = 0.004). Self-reported ease in undertaking mobility tasks over the 12-month period was not significantly different between the groups (0.49, 95% CI −0.91 to 1.90, p = 0.488).

**Conclusions:**

An individualised home exercise prescription significantly improved performance-based mobility but significantly increased the rate of falls in older people recently discharged from hospital.

**Trial Registration:**

Australian New Zealand Clinical Trials Registry ACTRN12607000563460

## Introduction

The development and widespread implementation of effective strategies to minimise disability and falls among older people is an urgent public health challenge due to the increasing proportion of older people in the global population. Older people recently discharged from hospital are at a particularly high risk of falls [1] and disability [2]. Previous studies have reported that 14% of older people fall in the first month after discharge from hospital [3] and 34% fall within three months of discharge from rehabilitation care [4].

It is well known that group- and home-based exercise programs can prevent falls in community-dwelling older people [5] but one previous trial found that a home exercise program of seated resisted knee extension exercises did not prevent falls in people who had recently been hospitalised [6]. Certain exercise programs have also been found to enhance balance and/or mobility in community-dwelling older people [7] and in those who have been in hospital [8]–[10]. As our previous meta-analysis [11] found greater effects on falls of exercise programs that challenge balance, a home exercise program that targets balance may prevent falls in people who have been in hospital. However, this has not been previously investigated.

We designed a simple exercise program that can be taught to older people, performed independently at home and individualised to ensure safety yet be sufficiently challenging. The program aimed to enhance balance and mobility and prevent falls by repetition of movements that are similar to daily activities such as standing up, walking, and stair climbing. We have previously found this program to improve mobility when used in a group-setting by stroke survivors [12] and in frail older people when used as part of a multi-disciplinary intervention [13], [14]. In this study, we sought to investigate the effects of this home-based exercise program on falls and mobility among older people who have had a recent hospital stay.

## Methods

### Trial design

A parallel pragmatic randomised controlled trial with equal allocation to intervention and control groups was conducted among 340 older people recently discharged from hospital. The protocol for this trial and supporting CONSORT checklist are available as supporting information; see Checklist S1 and Protocol S1.

### Ethical statement and trial registration

Ethical approval was obtained from the relevant human research ethics committees (University of Sydney, Northern Sydney Area Health Service, South Eastern Sydney Area Health Service and Hope Healthcare). When we commenced the trial we were aware of the importance of trial registration but not of the importance of registration prior to recruitment. Thus we recruited four trial participants prior to completing the trial registration process. The trial protocol was published [15].

### Participants

Participants were aged 60 years and over and had been admitted to and subsequently discharged from nine aged care, rehabilitation and orthopaedic wards at four public hospitals in Sydney, Australia. Potential participants were excluded if they: resided in a high-care residential facility (nursing home); had cognitive impairment (a Mini-Mental State Examination [16] score of less than 24); had insufficient English language to understand procedures; were unable to walk more than one metre even with an assistive device or the help of one person; or had a medical condition precluding a 12-month home exercise program (e.g. unstable cardiac disease or progressive neurological disease). Potential participants were approached about the study during their inpatient stay and their permission to be contacted after discharge from hospital was sought. Baseline assessments were conducted after participants had returned home, were medically fit to exercise, had completed hospital-initiated rehabilitation and provided written informed consent to study participation.

### Randomisation

Randomisation was stratified by hospital site and falls history (0–1 versus 2+ falls in the previous 12 months) using a computer-generated random number schedule with randomly ordered blocks of two, four and six. To ensure concealed randomisation to groups, the randomisation schedule was generated in advance by and only accessible to the first author who was not involved in participant recruitment, interviews or assessments. After completing the baseline assessment, and gaining informed consent and approval for trial participation from individual local medical officers, research staff contacted the first author by email to receive the group allocation for individual participants.

### Interventions

Three experienced physiotherapists delivered the intervention in participants' homes. Ten visits were scheduled over the 12-month study period, with more frequent visits at the beginning of the program to ensure safety and enable tailoring and progression of the program. Participants were asked to undertake a 20 to 30 minute program of lower limb balance and strengthening exercises up to six times per week for 12 months. The exercises were primarily conducted while standing and were based on the Weight-bearing Exercise for Better Balance program, which is available at www.webb.org.au. The physiotherapist prescribed the level of difficulty and number of repetitions for each exercise after an assessment of the participant's abilities. The Physical Activity Stage of Change model [17] was also used by the study physiotherapists to guide their approach to encouraging ongoing home-based exercise participation. Exercises that primarily targeted postural control (balance) included standing with a narrower base (aiming for tandem or single leg stance), forwards and sideways stepping/walking, and graded reaching activities in standing. The lower limb extensor muscle groups (i.e. hip and knee extensors and ankle plantar flexors), which act to prevent collapse of the lower limb, were targeted with exercises aiming to enhance muscle strength and control. Strengthening exercises included sit-to-stand, forward and lateral step-ups onto a small block, and heel raises in standing. Where appropriate, resistance for strengthening exercises was added by the use of weight-belts worn around the waist or weighted vests. The use of upper limbs to support the body while exercising was minimised but was advised where necessary to ensure safety. Participants were instructed on how to perform exercises with stable supports (such as a table) located nearby to hold onto if needed and, where appropriate, family members and/or carers were encouraged to assist with supervision of the exercise program. If a participant became unwell or had another admission to hospital, the program was resumed when the participant and the relevant professionals considered him/her well enough to participate again. The optimal intensity and type of exercises for each participant was re-assessed and adjusted by the study physiotherapists to ensure that the intervention remained challenging. Participants were not given any specific advice about general physical activity levels. Participants were provided with a booklet of safety precautions, instructions and photographs of exercises for use in exercise sessions at home. In addition, participants were provided with a logbook for recording exercises completed and effects of exercise (e.g. muscle soreness). The physiotherapists were asked to give their impression of the percentage of prescribed exercise that had actually been undertaken by each participant in the first, third, eighth and twelfth month of the intervention period. This rating was based on review of the exercise logs as well as discussion with the participant and carers present during the visit.

Participants in both groups received a 32-page education booklet about fall prevention. It included information about risk factors for falls, environmental modification for falls risk reduction and what to do after a fall but did not offer any specific advice about exercise. The control group did not receive any additional intervention as part of the study. Participants in both groups received usual care from health and community services. Participants did not commence in the study until they had completed all care from the admission during which they were recruited for the study. Thus usual care involved ongoing management of chronic conditions and routine management for any new conditions. This involved hospitalisation, visits to general practitioners, medical specialists, other health practitioners and Emergency Departments and home visits from community service providers to assist with daily tasks such as showering, dressing, meals and housework. Full details of health and community services used by trial participants will be published elsewhere (manuscript under review).

### Data collection

Data were collected from medical records, postal questionnaires, calendars, interviews and physical assessments. Information on medical history, diagnoses and medications at the time of recruitment were collected from medical records while participants were in hospital. Baseline assessments were conducted in participants' homes prior to randomisation. Further assessments were conducted three and 12 months after randomisation by physiotherapists and trained research assistants masked to group allocation. Participants were given 12 calendars and questionnaires about mobility at the baseline assessment and asked to record falls on the calendars and complete the questionnaires each month and return them in pre-paid envelopes to the research centre. Participants who did not return calendars or questionnaires were telephoned by a research assistant masked to group allocation to obtain the information. Participants who reported falling were also telephoned to obtain more information about the circumstances and consequences of such falls.

### Blinding

Staff who conducted interviews and assessments, received calendars and questionnaires, made phone calls and entered data were unaware of group allocation.

### Primary outcomes

The three primary outcome measures were fall rate, performance-based mobility and self-reported mobility [18].

1. Fall rate was calculated using the information provided on the monthly calendars described above. A fall was defined as an event in which the person unintentionally came to rest on the ground or other lower level which was not as a result of a violent blow, loss of consciousness, sudden onset of paralysis as in a stroke or an epileptic seizure [19].
2. Performance-based mobility was measured at 12 months after randomisation using the performance-based Short Physical Performance Battery [20], which involves the timed performance of three mobility tasks: the ability to stand for up to 10 sec with feet position in three different positions (together side by side, semi-tandem and tandem), 4-metre walk and time to rise from a chair five times. The primary analysis was conducted using the continuously scored lower extremity Summary Performance Score version [21] of this tool.
3. Self-reported level of ease in performing eight mobility tasks was measured monthly on a five-point scale and the scores summed. The eight mobility tasks were; getting in and out of bed, sitting up in bed, walking in the home, bending, walking half a mile and walking up and down stairs, walking 1 mile and undertaking moderate physical activity [18].

### Secondary outcomes

Secondary outcome measures were: additional measures of mobility; falls and risk of falling; strength; flexibility; quality of life; falls self-efficacy; health system and community service contact; assistance required from others; difficulty with daily tasks; community participation; and physical activity levels. These measures aimed to enhance understanding of the effects of the exercise program on multiple factors that contribute to an older person's functioning and quality of life.

Additional measures of mobility included: the Maximal Balance Range test (participants' ability to lean as far forward and backwards as possible); the Coordinated Stability test (participants' ability to adjust the body position in a steady and coordinated manner when near the limits of their base of support) [23]; the Step Test (stepping onto and off a 15 cm block as many times as possible in 15 seconds); and the time the participant could stand unsupported in single leg stance; the number of steps required to make a 180 degree turn. Falls were further assessed by comparing the proportion of people experiencing one or more falls over the 12-month follow-up period in the intervention and control groups. Risk of falling was assessed using the Physiological Profile Assessment [22] and choice stepping reaction time tests. The Physiological Profile Assessment provides a composite measure of risk of falling and includes measures of knee extension strength (measured with a spring balance with the participant sitting with the hip and knee at 90 degrees of flexion), postural sway, lower limb proprioception, reaction time and visual contrast sensitivity. Choice stepping reaction time has been found to be a composite measure of risk of falling when assessed with an electronic device [24]. In a modified version of the test, participants stood on a nonslip black mat (0.8 m×1.2 m) marked with four rectangular panels (32 cm×13 cm), one in front of each foot and one to the side of each foot. Participants were instructed to step onto specific rectangle panels in sequence as quickly as possible, using the left foot only for the two left panels (front and side) and the right foot only for the two right panels. The time for the entire routine was recorded in seconds. Grip strength was assessed using a portable dynamometer (JAMAR Hydraulic Hand Dynamometer manufactured by Sammons Prestons). Ankle range of motion was measured in a torque-controlled manner using a modification of the Lidcombe template procedure [25] in which the participant is seated and an inclinometer is used to assess changes in ankle angle. Quality of life was assessed using the physical and mental components of the SF 12 Version 2 and the EQ-5D [26] and a utility score was calculated using the SF6D and the EQ-5D. Fall-related self-efficacy was assessed using the Falls Efficacy Scale-International [27] in which level of concern about falling when carrying out a range of activities is rated on a 4-point scale. Participants were also asked about the need for assistance from others and community participation. Physical activity levels were assessed with the Incidental and Planned Exercise Questionnaire [28].

### Adverse events

Adverse effects of the exercise program were monitored using exercise diaries kept by participants. Participants in the intervention group were also advised to telephone study staff if they experienced any major adverse effects of the exercise program (defined as chest pain or muscle soreness lasting for more than 48 hours and interfering with daily activities or requiring medical attention).

### Sample size

We anticipated that the intervention would reduce the rate of falling. Sample size calculations indicated that 350 participants (175 per group) would be required for 80% power to detect as significant a 30% reduction in the rate of falling (i.e. an IRR of 0.70 using negative binomial regression analysis) in the 12-month follow-up period (alpha = 5%, two-sided). An 11-month average follow up period was used in the sample size calculation to account for loss to follow-up. This sample size would also be sufficient to detect clinically- and statistically- significant 10% between-group differences in lower extremity Summary Performance Scores and the self-reported mobility measure (power = 80%, p = 0.05, dropouts = 15%, alpha = 5%, two-sided).

### Statistical methods

Analyses were conducted according to the pre-defined statistical analysis plan on an intention-to-treat basis and were adjusted only for baseline score except where otherwise indicated. All statistical tests were two-sided and p-values were considered significant when their values were less than 0.05. All analyses were undertaken using Stata software (StataCorp LP, College Station, Texas, versions 12 and 13). The number of falls per person-year was analysed using negative binomial regression to estimate the difference in fall rates between the two groups (primary outcome). Length of follow-up was included as an exposure term in these models, i.e. the logarithm of the days of follow-up was added as an offset. A blind review by the statistician (GH) prior to analysis revealed that the negative binomial model was appropriate. Secondary analyses were also conducted to compare the proportion of fallers in the two groups (using modified Poisson regression models) and to compare the group rates of indoor falls, falls requiring health care intervention, falls in the four 3-month periods of the study. For the performance-based mobility measure (lower extremity Summary Performance Score) linear regression models were used to compare the groups on three-month (secondary outcome) and 12-month (primary outcome) assessment scores adjusted for baseline scores. This approach was also used for the continuously scored secondary outcome measures. Where outcome variables were markedly skewed the difference between initial and final scores was used in the models instead of the final scores. For the self-reported mobility measure, analyses were conducted by comparing the average of the 12 monthly scores between the groups (primary outcome). Negative binomial regression was also used for other count variables such as the numbers of health service contacts and social outings. Ordinal regression was used for the health status question from the EQ-5D. In cases where individuals were unable to carry out a test due to physical impairments, a score of 0 was given for variables where low score indicates poor performances and a score of the mean plus three standard deviations was allocated for variables where high score indicates poor performances.

Pre-planned subgroup analyses were conducted using interaction terms in the models to assess whether the intervention had differential effects in those with 2 or more falls in the year prior to study recruitment (versus 0 or 1 falls), those with poorer cognition (above and below the pre-specified cut-point of the median MMSE score, which was 28) and slower baseline walking speed (as a continuous interaction term). The interaction between group and baseline walking speed for the falls outcome was graphed using R software. Further exploratory analyses were undertaken to investigate whether any differential effects of the intervention on falls were evident on the basis of other baseline characteristics. Secondary analyses tested the impact of adjusting for the stratification variables. Secondary per protocol analyses were also conducted and included only those participants who completed (i) at least 50% and (ii) at least 80% of prescribed interventions. In order to lessen the risk of “confounding by indication” (i.e., the likely tendency for people who are able to do more exercise to have better outcomes) in this analysis, outcomes in intervention group participants who undertook more than 50% and 80% of prescribed sessions were then compared with outcomes in the control group after adjusting for likely predictors of outcome (baseline mobility, age, fear of falling, co-morbidity, past falls, PPA fall risk score and cognition).

Additional secondary analyses used instrumental variable regression to estimate the complier average causal effect [29],[30]. Participants in the intervention group were considered to have adhered to the exercise program if they completed at least 80% of the exercise program, and it was assumed that participants in the control group could not access the intervention. This analysis was conducted on the two primary mobility outcomes, but not on the falls outcome because the exclusion restriction assumption made in instrumental variable regression [29] was considered less tenable for the falls outcome.

## Results

### Participant flow

**Figure 1:**
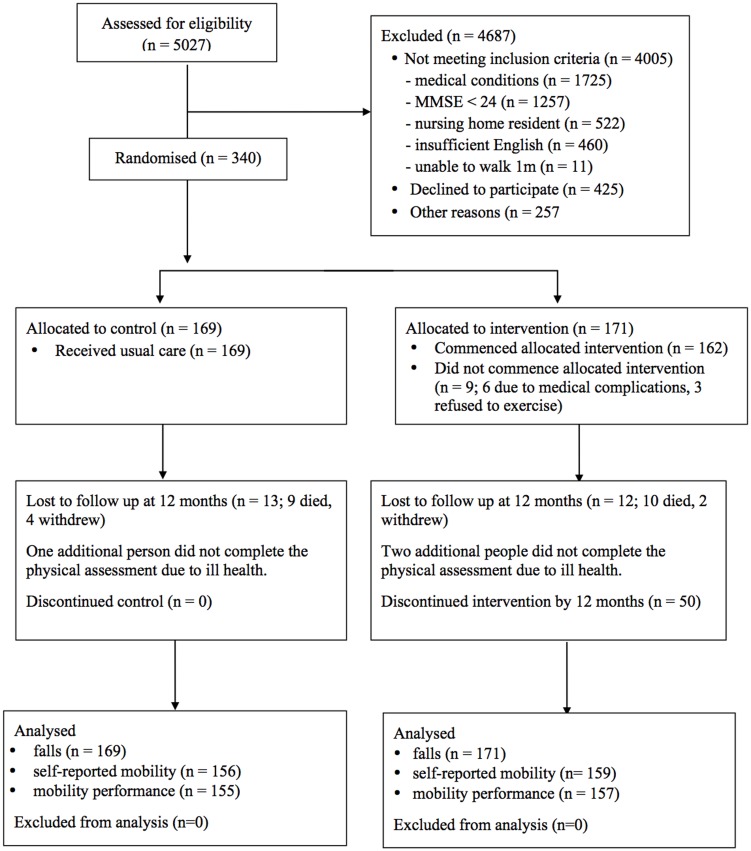
Participant flow.

Figure 1 shows the flow of participants through the study. Three hundred and forty people were randomised to one of the two groups; 171 to the intervention group and 169 to the control group. Baseline assessments were conducted a median of 62 days (IQR 53) days after hospital discharge. All participants provided at least one month of falls data so were included in the falls analysis. Twelve months of falls data were available for 320 people (94% of those randomised; 159 from the control group and 161 from the intervention group). The average length of falls follow up was 355 (SD 47) days in the exercise group and 354 (SD 51) days in the control group. The 12-month assessment was completed by 312 participants (92% of those randomised) and was undertaken an average of 374 (SD 12) days after the baseline assessment. Twelve months of monthly self-reported mobility data were available for 315 participants (93% of those randomised).

### Recruitment

Participant recruitment was undertaken between November 2007 and February 2011. Recruitment was stopped ten participants prior to the original target due to exhaustion of study funds. Follow up was completed in February 2012.

### Baseline data

Participants had an average age of 81.2 years (SD 8.0), an average of 6.8 health conditions (SD 2.7), and were prescribed an average of 7.5 medications (SD 3.3). Seventy-four per cent were women and 70% had fallen in the past year. Table 1 provides a summary of the baseline demographics of the study sample and describes the primary diagnoses at hospital admission. Table 2 outlines participant answers to interview questions and performance in the physical assessments at baseline. As the tables show, there were no marked differences between the groups at baseline.

**Table 1:** Baseline characteristics of participants.

Characteristic	Control (n = 169)	Intervention (n = 171)
Age, mean (SD) years	80.8 (8.2)	81.6 (7.8)
Hospital length of stay, mean (SD) days	15.6 (15.7)	17.0 (19.3)
Mini Mental State Examination score, mean (SD)/30	28.0 (1.8)	27.9 (2.0)
Females, n (%)	128 (76)	123 (72)
1 or more falls in previous 12 months, n (%)	117 (69)	123 (72)
Medications, mean (SD) number	7.5 (3.3)	7.5 (3.3)
Psychotropic medication, n (%)	30 (18)	17 (10)
Benzodiazepine medication, n (%)	31 (18)	20 (12)
Living in low-care residential facility (hostel), n (%)	12 (7)	8 (5)
Co-morbidities, mean (SD) number	6.9 (2.7)	7.2 (2.9)
Primary diagnosis on admission (from medical file) n (%)		
Lower limb fracture	24 (14)	26 (15)
Upper limb fracture	7 (4)	7 (4)
Vertebral fracture	9 (5)	12 (7)
Respiratory illness	13 (8)	11 (6)
Infection	13 (8)	13 (8)
Fall and soft tissue damage	15 (9)	19 (11)
Neurological condition	2 (1)	4 (2)
Musculoskeletal condition	21 (12)	19 (11)
Cerebrovascular disease	4 (2)	4 (2)
Ischaemic heart disease	3 (2)	2 (1)
General medical conditions	11 (7)	12 (7)
General symptoms	14 (8)	12 (7)

**Table 2:** Primary mobility outcomes and secondary outcomes.

	Control			Intervention			Difference between groups (baseline adjusted)	
	Baseline, n = 169	3 month, n = 137	12 month, n = 155	Baseline, n = 171	3 month, n = 156	12 month, n = 157	3month mean (95% CI), p	12 month mean (95% CI), p
**Mobility**								
Summary Performance Score (primary outcome), 0–3, mean (SD)^a^^, ^ c	1.92 (0.48)	1.98(0.53)	1.94(0.56)	1.92 (0.46)	2.05(0.48)	2.06(0.52)	*0.07 (−0.006 to 0.15), p = 0.070*	**0.13 (0.04 to 0.21), p = 0.004**
Mobility task ease over 12 months (primary outcome), 0–40, mean (SD)^a^^, ^ d	28.2 (7.2)		27.7 (6.7), n = 156	27.6 (6.7)		28.2 (6.0), n = 159		0.49, 95% CI −0.91 to 1.90, p = 0.488
Short Physical Performance Battery, 0–12, mean (SD)^a^^, ^ d	6.73 (2.84)	7.14 (2.96)	6.89 (1.92)	6.72 (2.73)	7.48(2.94)	7.41(2.02)	*0.39 (−0.02 to 0.80), p = 0.065*	**0.50 (0.11 to 0.90), p = 0.013**
Sit to stand, stands/sec, mean (SD)^a^^, ^ c	0.22(0.16)	0.23 (0.16)	0.22 (0.2)	0.22 (0.16)	0.26(0.17)	0.28 (0.2)	0.02 (−0.009 to 0.04), p = 0.211	**0.05 (0.02 to 0.84), p = 0.000**
Gait speed, m/sec, mean (SD)^a^^, ^ d	0.72 (0.3)	0.74 (0.32)	0.76(0.32)	0.74 (0.3)	0.75(0.30)	0.77(0.31)	−0.007 (−0.05 to 0.049), p = 0.978	−0.011 (−0.06 to 0.04), p = 0.662
Standing Balance, sec, 0–30, mean (SD)^a^^, ^ c	23.3 (7.1)	24.1 (7.7)	23.4 (8.3)	22.9(7.4)	24.9 (7.0)	24.4 (7.5)	1.04 (−0.36 to 2.44), p = 0.144	1.18 (−0.30 to 2.7), p = 0.117
Single leg stance time, sec, 0–10, mean (SD)^a^^, ^ c	2.2 (3.7)	3.0 (4.1)	3.0 (4.1)	2.2 (3.8)	3.5 (4.2)	3.2 (4.3)	0.32 (−0.42 to 1.07), p = 0.389	0.15 (−0.56 to 0.87), p = 0.677
Maximum Balance Range, mm, mean (SD)^a^^, ^ d	115.49(40.91)	114.94(47.3)	115.8(47.6)	111.19(41.87)	120.7(47.0)	122.5(50.3)	**10.16 (1.84 to 18.47), p = 0.017**	*8.06 (−0.46 to 16.6), p = 0.064*
Coordinated Stability, score, mean (SD)^b^^, ^ c	21.77(12.64)	19.9 (13.2)	21.7 (15.3)	24.44 (13.82)	22.78(13.7)	22.8 (13.6)	0.76 (−1.44 to 2.95), p = 0.498	−0.395 (−2.81 to 2.02), p = 0.747
Step Test, steps, mean (SD)^a^^, ^ c	7.4 (4.9)	8.5 (5.4)	7.9 (5.9)	7.4 (4.8)	8.9 (5.3)	8.7 (6.0)	0.36 (−0.36 to 1.09), p = 0.329	0.69 (−0.14 to 1.51), p = 0.101
Steps required to turn 180 degrees, mean (SD)^b^^, ^ d	5.6(1.8)	5.5(1.9)	5.6(2.2)	5.9(2.6)	5.3(1.7)	5.3(2.3)	−0.29 (−0.64 to 0.06) p = 0.110	**−0.43 (−0.82 to −0.05), p = 0.026**
**Fall risk**								
PPA Score, mean (SD)^b^^, ^ d	1.20 (1.41)	1.41 (1.59)	1.59 (1.64)	1.37 (1.49)	1.42 (1.50)	1.61 (1.75)	−0.14 (−0.40 to 0.11), p = 0.269	− 0.45 (−0.32 to 0.23), p = 0.745
Postural sway on foam total length, mean (SD)^b^^, ^ c	157.5(99.5)	168.6(101.1)	180.1(107.9)	170.4 (99.5)	161.9(93.7)	167.3(100.0)	**−20.36 (−38.03 to −2.68), p = 0.024**	*−18.03 (−36.58 to 0.52), p = 0.057*
Knee extension strength, kg, mean (SD)^a^^, ^ d ^, ^ g	14.6 (6.1)	13.2 (6.1)	13.3(5.8)	14.6 (6.4)	13.8 (7.0)	14.0(6.8)	0.20 (−1.00 to 1.39), p = 0. 745	0.61 (−0.49 to 1.72), p = 0.275
Hand Reaction time, msec, mean (SD)^b^^, ^ c	310.9 (104.6)	333.7(146.1)	334.3(132.7)	307.5 (108.1)	318.3(122.2)	346.9(149.3)	−11.82 (−38.82 to 15.17), p = 0.389	17.48 (−11.25 to 46.20), p = 0.232
Choice Stepping reaction time, step/sec, mean (SD)^a^^, ^ c	0.20 (0.13)	0.22 (0.14)	0.21 (0.2)	0.20 (0.13)	0.23 (0.15)	0.22 (0.2)	0.01 (−0.02 to 0.03), p = 0.591	0.01 (−0.02 to 0.03), p = 0.585
**Grip strength**, kg, mean (SD)^a^^, ^ d	20.4 (8.2)	19.99 (8.83)	19.05(7.99)	20.8 (8.9)	20.92 (8.91)	19.61(8.67)	0.48 (−5.07 to 1.40), p = 0.337	0.37 (−0.58 to 1.33), p = 0.444
**Ankle flexibility**, score, mean (SD)^a^^, ^ d	24.53 (9.54)	26.15(10.68)	24.98(9.62)	25.09 (10.49)	25.21(11.26)	25.08(10.99)	−1.19 (−3.74 to 1.36), p = 0.359	0.014 (−2.29 to 2.33), p = 0.991
**Quality of life**								
Health compared with past 12 months, n (%)^e^							OR 1.11, (0.72 to 1.72), p = 0.642	**OR 1.81 (1.19 to 2.76), p = 0.006**
Better	46 (27)	38 (28)	37 (24)	30 (18)	44 (29)	60 (38)		
Much the same	69 (41)	48 (35)	69 (45)	59 (35)	50 (33)	60 (38)		
Worse	54 (32)	50 (37)	49 (32)	82 (48)	59 (39)	37 (24)		
EQ5D utility score, mean (SD)^a^^, ^ c	0.62(0.23)		0.60(0.33)	0.63(0.23)		0.66(0.27)		0.04, −0.02 to 0.10 p = 0.229
SF12 Physical Component, mean (SD)^a^^, ^ d	38.17 (8.36)	40.32 (9.04)	39.27 (9.26)	37.44 (8.90)	40.10 (8.53)	40.37 (8.29)	−0.10 (−1.83 to 1.64), p = 0.914	*1.45 (−0.24 to 3.14), p = 0.093*
SF12 Mental Component, mean (SD)^a^^, ^ d	54.70 (6.79)	54.18 (7.01)	55.19 (7.09)	54.71 (6.49)	55.31 (6.37)	55.87 (5.02)	1.06 (−0.38 to 2.5), p = 0.149	0.70 (−0.59 to 1.99), p = 0.288
SF6D utility score, mean (SD)^a^^, ^ d	0.67 (0.10)	0.69 (0.12)	0.70 (0.12)	0.66 (0.10)	0.70 (0.11)	0.71 (0.11)	0.01 (−0.01 to 0.03), p = 0.346	*0.02 (−0.004 to 0.04), p = 0.096*
**Falls efficacy scale**, 7–28, mean (SD)^b^^, ^ c	11.56 (4.18)	11.97 (3.82)	13.14(4.87)	11.70 (4.09)	11.69 (3.94)	12.57 (4.22)	−0.31 (−1.03 to 0.41), p = 0.393	−0.52 (−1.40 to 0.36), p = 0.243
**Health and community services**, mean/month (SD)^b^^, ^ f	5.5 (6.7)		3.6 (6.1)	4.5 (5.0)		3.1 (7.4)		IRR 1.09 (0.85 to 1.40), p = 0.482
**Assistance** occasions/month, mean (SD)^b^^, ^ f	37.3 (43.2)		24.7 (37.5)	40.2(42.9)		25.3 (36.9)		IRR 0.91 (0.62 to 1.33), p = 0.631
**Ease of personal care tasks**, 0–50, mean (SD)^a^^, ^ c	41.4 (7.4)		38.7 (10.3)	41.5 (7.3)		40.5 (8.6)		*1.5 (−0.26 to 3.33), p = 0.094*
**Community participation** outings/month, mean (SD)^a^^, ^ f	5.9(7.3)	7.6 (10.3)	6.0(8.2)	5.8 (8.3)	6.2 (8.0)	7.5 (9.7)	0.91 (0.69 to 1.18), p = 0.459	**IRR 1.35 (1.05 to 1.75), p = 0.024**
**Physical activity**								
Total Physical Activity Score, hours/week^a^^, ^ c	18.13 (11.56)	26.26(15.74)	29.29(21.07)	18.5 (12.98)	27.34(16.34)	30.89(20.11)	0.59 (−2.46 to 3.64), p = 0.703	1.05 (−2.89 to 4.99), p = 0.600
Home Exercise, hours/week^a^^, ^ c	0.15 (0.33)	0.71 (1.30)	0.45 (0.98)	0.22 (0.47)	0.87 (1.51)	0.81 (1.40)	0.10 (−0.22 to 0.42), p = 0.521	**0.30 (0.03 to 0.58), p = 0.029**

a Higher scores reflect better performance.

b Lower scores reflect better performance.

c Between group differences are from linear regression models with change score (final minus initial) score as the dependent variable and group allocation and baseline performance on that item as independent variables.

d Between group differences are from linear regression models with follow-up score as the dependent variable and group allocation and baseline performance on that item as independent variables.

e Between group differences are from ordinal regression models with follow-up score as the dependent variable and group allocation and baseline performance on that item as independent variables.

f Between group differences are from negative binomial regression models with the total number of events as the dependent variable and group allocation and number of events at baseline as independent variables.

g some participants unable to be assessed due to knee pain or skin problems; data available for 168 per group at baseline; 3 months 131 control, 152 intervention;12 months 151 control, 156 intervention.

Bold indicates p<0.05 for between group differences. Italics indicate p<0.1 for between group differences.

### Intervention delivery

The intervention was offered to all 171 participants randomised to the intervention group but nine did not commence a home exercise program due to new health problems (n = 6) or refusal to exercise (n = 3). Participants who commenced the program received an average of 9.2 (SD 1.7) home visits. At the start of the study, participants agreed to undertake an average of 6 exercise sessions (range 3 to 7) involving an average of 5 different exercises (range 2 to 8) and an average of 59 exercise repetitions per week (SD 39). In the first month, participants reported completing an average of 5 sessions (range 1 to 7) and study physiotherapists estimated that 77% of prescribed repetitions were completed (SD 34). At the end of 12 months, 103 people (61% of the intervention group) continued to exercise and, among exercisers, the average number of exercise sessions completed was 4 (range 1 to 7). Table 3 provides further details of intervention dose and adherence.

**Table 3:** Intervention dose prescribed and adherence over the 12 month study period.

Time point	Participants who agreed to exercise, number (% of 171)	Exercise sessions agreed to, median (range)	Different exercises prescribed, median (range)	Exercise repetitions agreed to per session, mean (SD)	Participants who actually exercised, number (% of 171)	Exercise sessions completed, median (range)	Physiotherapist's estimate of % prescribed exercise completed, mean (SD)	Physiotherapist's estimate of number of exercise repetitions completed, mean (SD)
Month 1	162 (95%)	6 (0 to 7)	5 (0 to 8)	59 (39)	153 (90%)	5 (0 to 7)	77 (34)	266 (227)
Month 3	151 (88%)	5 (0 to 7)	6 (0 to 9)	93 (65)	138 (81%)	4 (0 to 7)	64 (38)	391 (396)
Month 8	130 (76%)	3 (0 to 7)	6 (0 to 9)	77 (70)	113 (66%)	3 (0 to 7)	51 (43)	289 (352)
Month 12	121 (71%)	3 (0 to 7)	5 (0 to 10)	72 (71)	103 (60%)	3 (0 to 7)	47 (43)	250 (335)

### Falls

During the 12-month study period, 168 people (49% of participants) reported 300 falls (Table 4). The intervention group reported a higher fall rate (177 falls, 1.0 fall per person, SD 1.23) than the control group (123 falls, 0.73 falls per person, SD 1.22) and this difference (primary outcome) was statistically significant (incidence rate ratio 1.43, 95% CI 1.07 to 1.93, p = 0.017, n = 340). The estimate of effect of the intervention on rate of falls was not changed by adjustment for the stratification variables i.e., study site and past falls (IRR 1.40, 95% CI 1.05 to 1.87, p = 0.021). There was also a higher proportion of fallers in the exercise group (98 people, 57%) than the control group (70 people, 41%) and this difference was statistically significant (RR 1.38, 95% CI 1.11 to 1.73, p = 0.004). Indoor falls were more frequent in the intervention group than in the control group (IRR 1.62, 95% CI 1.13 to 2.33, p = 0.009) but there was no significant difference in outdoor falls (IRR 1.10, 95% CI 0.71 to 1.69, p = 0.670), falls requiring health care intervention (IRR 1.14, 95% CI 0.76 to 1.73, p = 0.524) or fall-related fractures (15 in control group, 14 in intervention group). The increase in falls in the intervention group was particularly evident in the first and second 3-month periods of the study and was statistically significant in the second 3-month period. A loss of balance was the most common participant reported reason for falling (127 falls) followed by a trip (93 falls). No falls were reported while undertaking the home exercise program.

**Table 4:** Falls outcomes.

	Control (n = 169)	Intervention (n = 171)	Difference between groups
Falls per participant, n (%)			
0	99 (59)	73 (43)	1.38 (1.11 to 1.73), p = 0.004^a^
1	45 (27)	58 (34)	
2	12 (7)	16 (9)	
3	4 (2)	13 (8)	
≥4	9 (5)	11 (6)	
All falls (primary outcome)	123	177	1.43 (1.07 to 1.93), p = 0.017^b^
Fall location			
Indoors	79	128	1.62 (1.13 to 2.33), p = 0.009^b^
Outdoors	44	49	1.10 (0.71 to 1.69), p = 0.670^b^
Falls with injuries			
Received health care	53	61	1.14 (0.76 to 1.73), p = 0.524^b^
Fractures	15	14	n/a^c^
Falls by time period			
Months 0–3	27	44	1.61 (0.98 to 2.65), p = 0.061, n = 340^b^
Months 4–6	19	49	2.56 (1.42 to 4.62), p = 0.002, n = 337
Months 7–9	27	25	0.92 (0.52 to 1.62), p = 0.774, n = 327
Months 9–12	50	59	1.16 (0.75 to 1.78), p = 0.503, n = 323
Participant reported cause of fall			
Lost balance	50	77	
Legs gave way	11	14	
Trip	35	58	
Slip	14	17	
Other	13	11	
Falls by gait subgroup			
≥ 0.73 m/s at baseline	80	85	1.95 (1.28 to 2.99), p = 0.002, n = 167
<0.73 m/s at baseline	43	92	1.15 (0.76 to 1.75), p = 0.498, n = 173

a Relative risk, 95% confidence interval and p value from modified Poisson regression model for the proportion of participants who had one or more falls in the 12 month follow up period in the intervention group compared to the control group.

b Incidence rate ratio, 95% confidence interval and p value from negative binomial regression model comparing the number of falls in the 12 month follow up period in the intervention group compared to the control group.

c Incidence rate ratio not calculated due to small numbers.

### Mobility (primary outcomes)

At 12 months, performance-based mobility (Lower Extremity Summary Performance Score, range 0–3) improved significantly more in the intervention group (mean 2.06, SD 0.52) than in the control group (mean 1.94, SD 0.56, between group difference in change score after adjusting for baseline performance 0.13, 95% CI 0.04 to 0.21, p = 0.004). Self-reported ease in undertaking mobility tasks over the 12-month period (range 0–40) was not significantly different between the groups (0.49, 95% CI −0.91 to 1.90, p = 0.488, Table 2).

### Sub-group analyses for the primary outcomes

**Figure 2:**
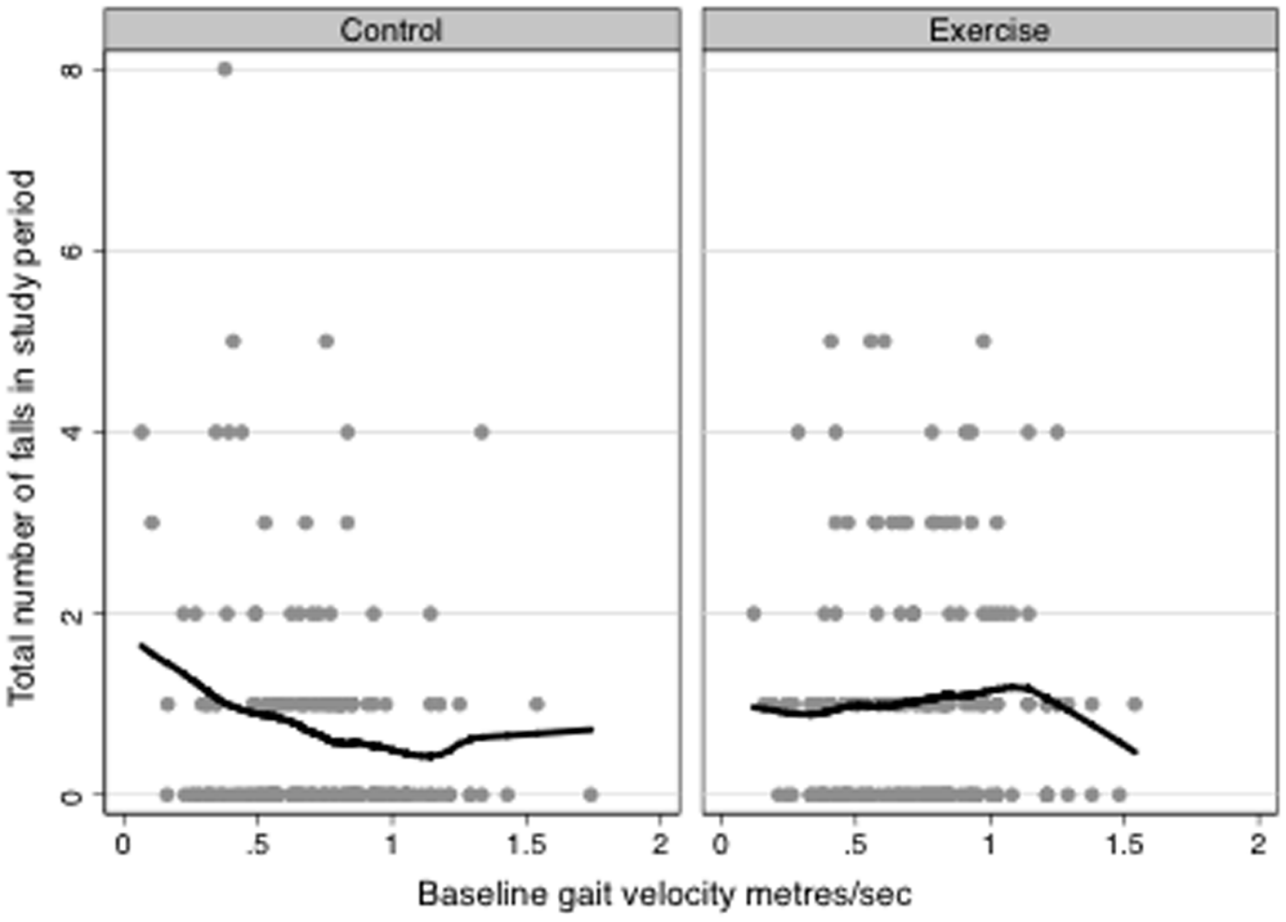
Number of falls in the 12-month study period by baseline walking velocity and group. The lines shown are locally weighted regression lines using Stata's “lowess” command.

Pre-planned subgroup analyses found that the increase in falls was greater in participants who walked more quickly at baseline (mean gait speed at baseline 0.73 m/sec, interaction term p = 0.014, see Figure 2 for the graph of the interaction) but there was no evidence of differential effects on falls among those with two or more past falls or lower MMSE scores (<28). Given the unexpected finding for the falls outcome, further exploratory sub-group analyses were undertaken. These did not indicate differential intervention impacts on the basis of gender (p for interaction = 0.86), age (p = 0.88), co-morbidity (p = 0.69), falls efficacy (p = 0.26), walking aid use (p = 0.33), recruitment source (rehabilitation versus acute hospital, p = 0.23), PPA risk of falling score (p = 0.52), or postural sway (p = 0.24) but found trends towards a greater increase in falls among people with better knee extensor strength (p = 0.08) in those with falls as a presenting condition on admission to hospital (p = 0.12) and in those with a self-reported current or previous neurological condition (p = 0.18).

There was a greater impact on performance-based mobility for those with better MMSE scores (p for interaction = 0.037; between-group difference in those with MMSE scores less than 28 −0.001, 95% CI −0.16 to 0.16, p = 0.995, n = 111; between-group difference in those with MMSE scores 28 to 30 0.19, 95% CI 0.09 to 0.29, p<0.001, n = 201). There was no evidence for differential effects on the performance-based mobility outcome on the basis of baseline gait speed (p for interaction = 0.626) or past falls (p for interaction = 0.274). There was no evidence for differential effects of the intervention on the self-reported mobility outcome on the basis of baseline gait speed (p for interaction = 0.254), MMSE score (p for interaction = 0.589) or past falls (p for interaction = 0.256).

### Secondary outcomes

Secondary outcome results are shown in Table 2. The three-month assessment was completed by 292 participants (86% of those randomised) and was undertaken an average of 103 (SD 14) days after the baseline assessment. At 3 months after randomisation, there were significant between group differences for standing balance (maximal balance range and postural sway on foam) that favoured the exercise group after adjustment for baseline performance. There were no significant between-group differences for any other secondary outcomes at 3 months.

At 12 months after randomisation, there were significant between group differences in the 12-item version of the Short Physical Performance Battery, sit-to-stand ability, turning ability, reported health status (from the EQ-5D), social outings and reported home exercise that favoured the exercise group after adjustment for baseline performance. There were no significant between-group differences for any other secondary outcomes at 12 months. There were trends (p<0.1) for better outcomes in the intervention group in measures of balance (maximal balance range and postural sway), quality of life (physical component), ease of daily task performance and community participation.

### Adverse events

Twelve intervention group participants reported adverse events that may have been associated with the physical assessment or exercise program that resulted in limitations of activity of daily living for at least 48 hours or required medical attention. These included finger pain following the grip strength assessment in two people, thigh pain after the assessment in one person, low back pain in five people, calf pain in one person, knee pain in one person and exacerbation of hernia symptoms in two people. For 27 other people pre-existing conditions (mainly musculoskeletal) limited progression of the exercises.

### Associations between intervention adherence and outcomes

The complier average causal effects of intervention estimated with instrumental variable regression were, inevitably, larger than the intention to treat-based estimates of effect. In compliant participants, exercise increased performance-based mobility by a mean of 0.27 points (95% CI 0.09 to 0.45) and increased self-reported ease of mobility by a mean of 1.1 points (95% CI −2.0 to 4. 2). As with the primary analysis, the effect on performance-based mobility was statistically significant (p = 0.003) but the effect on self-reported ease of mobility was not (p = 0.485).

## Discussion

This study found that a physiotherapist-prescribed tailored home exercise program enhanced performance-based mobility but significantly increased falls in older people recently discharged from hospital. Planned sub-group analyses revealed a greater increase in falls in those who walked more quickly at baseline and a greater improvement on performance-based mobility in those with better MMSE scores. The impact on mobility was greater in those who adhered well to the intervention.

The impact of the intervention on the continuously-scored primary measure of performance-based mobility was approximately 7% of baseline values (i.e., less than the 10% between-group difference we considered to be clinically meaningful prior to undertaking the study) yet represented a 0.5 point between-group difference in the 12-point version of the Short Physical Performance Battery a difference previously suggested to be “meaningful” [31]. This positive impact on mobility needs to be interpreted in the context of the increase in falls, which at 40% would probably be considered to be clinically meaningful by most.

The strengths of this study are its relatively large sample size, pragmatic design using a program that could be delivered as part of routine care, broad inclusion criteria and use of methods designed to reduce the risk of bias such as concealed random allocation to groups, blinded outcome assessment, small loss to follow up and pre-planned statistical analyses. The study's primary limitations are the commonly-reported sub-optimal adherence to the home exercise intervention (29% of those randomised to the intervention group no longer exercising at 12 months), the (unavoidable) reliance on self-reported falls data and the large number (93%) of screened participants who did not participate in the study which means that the sample may not be representative of older people recently discharged from hospital. The intention to treat approach used for the primary analyses probably underestimates the impact of the intervention in those who undertake a higher dose of ongoing exercise. A more supervised exercise program may have had different impact as it would have enabled ongoing encouragement and tailoring of exercise as well as the safe delivery of more challenging exercise.

These results differ from previous trials and meta-analyses showing fall prevention effects from home exercise interventions [5]. However, previous studies showing home exercise to prevent falls have been conducted in healthier older people than our sample. For example, the studies of the Otago Exercise Programme were conducted in people recruited from general practice records rather than those seeking health care [32]. Participants in the two Otago trials [33], [34] were prescribed an average of 2–3 medications whereas participants in our trial were prescribed an average of 7 medications. As the WEBB program used in the present study included several exercises that also form part of the Otago Exercise Programme it seems unlikely that the use of a different program explains the different result in this study. Rather these results suggest that home exercise is not an appropriate single fall prevention intervention for people who have been recently hospitalised and this finding may apply to other high-risk populations. Our meta-analysis found trials of exercise conducted in higher risk samples to have smaller fall prevention effects than those conducted in the general population [11], [35]. For example, one trial found a non-significant increase in falls from the Otago Exercise Programme in people with visual impairment [36] and another found a non-significant increase in falls from a group exercise program, especially in frail older people [37]. Conversely a highly supervised group exercise intervention did prevent falls in people with multiple previous falls [38] indicating uncertainty in this field.

Possible explanations for the greater number of falls in the intervention group include; i) harmful impact of exercise due to fatigue, exercise-induced muscle soreness or other pain exacerbated by exercise; ii) increased confidence or exposure to physical activity due to enhanced mobility; iii) harmful impact of partial adherence to the intervention (as outcomes were better in adherers after adjusting for other factors); iv) different time course of improvements in mobility and falls in this population (as the increase in falls was most evident in the first six months of the intervention period but the increase in mobility was greater at 12 months than 3 month assessments); v) over-reporting by the intervention group due to an increased awareness of falls; and vi) chance. Further research in high-risk populations is needed to explore these possibilities. The significant subgroup finding that the increase in falls was primarily among intervention group participants who walked more quickly at baseline should be treated with caution but warrants further investigation. It is possible that the faster walkers were actually walking “too quickly” to be safe given their recent illnesses and that this was somehow exacerbated by the intervention. The differential impact of the intervention on the mobility outcomes suggests that the more cognitively able and those with fewer past falls benefit more from this home exercise intervention in terms of mobility.

The impact of interventions specifically targeting safety (such as occupational therapy interventions), multifactorial programs and supervised exercise programs in people after recent hospital stays warrants investigation. Self-efficacy fall prevention interventions (such as the Stepping On program) [39] have been found to prevent falls in the general older population and also warrant investigation in older people following a hospital stay. Further research could also further investigate the interaction between increased mobility, physical activity, cognition, behaviour, and falls in high-risk people.

In conclusion, this approach to home exercise prescription significantly improved performance-based mobility but significantly increased the rate of falls in people after recent hospital stays. These findings suggest, therefore, that this form of home exercise cannot be recommended as a fall-prevention intervention for this group. Further studies, including the evaluation of the impact of multifactorial programs, are required to identify effective programs for preventing falls in this high-risk population.

## Supporting Information

Checklist S1 **Consort Checklist.**
(DOC)

Protocol S1 **Trial Protocol.**
(PDF)
